# Using the Person-Based Approach to Develop a Digital Intervention Targeting Diet and Physical Activity in Pregnancy: Development Study

**DOI:** 10.2196/44082

**Published:** 2023-05-26

**Authors:** Alexandra Rhodes, Arya Pimprikar, Alison Baum, Andrea D Smith, Clare H Llewellyn

**Affiliations:** 1 Research Department of Behavioural Science and Health Institute of Epidemiology and Healthcare University College London London United Kingdom; 2 Best Beginnings London United Kingdom; 3 Medical Research Council Epidemiology Unit School of Clinical Medicine University of Cambridge Cambridge United Kingdom

**Keywords:** digital, app, dietary, physical activity, lifestyle, pregnancy, prenatal, person-based approach, behavior change habit formation, intervention

## Abstract

**Background:**

In pregnancy, eating well, keeping active, and avoiding excessive weight gain are associated with better maternal and fetal health outcomes. Dietary and physical activity (PA) interventions can be effective in changing behaviors and managing weight gain. The comparatively lower cost and greater accessibility of digital interventions make them an attractive alternative to in-person interventions. Baby Buddy is a free pregnancy and parenting app from the charity Best Beginnings. Designed to support parents, improve health outcomes, and reduce inequalities, the app is actively used within the UK National Health Service. It offers an ideal platform for delivering and evaluating a new prenatal dietary and PA intervention.

**Objective:**

The aim of this study was to create a theory-based intervention within Baby Buddy to empower, encourage, and support expectant parents to develop healthier dietary and PA habits for pregnancy and parenthood.

**Methods:**

The intervention’s development process was guided by the Behavior Change Wheel, with the person-based approach used to create and test its design. Three stages of qualitative research with pregnant and recently pregnant parents guided the intervention design. Study 1 (n=30), comprising 4 web-based focus groups and 12 telephone interviews, gauged response to the rudimentary concept and generated ideas for its development. Results were analyzed thematically. At this stage, the *guiding principles* for the intervention development were established, and regular team meetings ensured that the intervention design remained aligned with Best Beginnings’ objectives, evidence-based approach, and feasibility criteria. Study 2 (n=29), comprising web-based individual and couple interviews, explored design ideas using wireframes and scripts and generated iterative feedback on the intervention content, branding, and tone. A *table of changes analysis* tracked design amendments. Study 3 (n=19) tested an app prototype using *think-aloud* interviews with current Baby Buddy users. A patient and public involvement and engagement activity (n=18) and other expert contributors (n=14) provided ad hoc input into the research process and design development.

**Results:**

Study 1 confirmed the appeal and relevance of the intervention concept and its novel approach of including partners. The identified themes underpinned the development of the intervention design. Iterative feedback from study 2, in conjunction with patient and public involvement and engagement and expert contributor input, helped refine the intervention design and ensure its relevance and appeal to a diverse target user group. Study 3 highlighted functionality, content, and design issues with the app prototype and identified ways of improving the user experience.

**Conclusions:**

This study illustrates the value of combining a theoretical method for intervention development with the person-based approach to create a theory-based intervention that is also user-friendly, appealing, and engaging for its target audience. Further research is needed to evaluate the effectiveness of the intervention in improving diet, PA, and weight management in pregnancy.

## Introduction

### Background

Eating a nutritious and well-balanced diet during pregnancy is important for healthy fetal development and maternal well-being. Observational studies show that a diet high in fruit, vegetables, whole grain cereals, legumes, fish, and nuts and low in dairy products and red meat is associated with reduced risk of both gestational diabetes in mothers and cardiometabolic and congenital defects in offspring [1-3]. Conversely, poor quality of diet, independent of calorific content, is associated with increased risk of gestational diabetes, large-for-gestational-age babies, and infant adiposity [4-6]. Eating well and keeping active during pregnancy reduces women’s risk of excessive gestational weight gain (GWG), which is associated with adverse maternal and fetal health outcomes during pregnancy and at birth [7,8]. Excessive GWG also increases a woman’s risk of postpartum weight retention and her chances of starting a subsequent pregnancy with overweight or obesity [9]. Moreover, there is growing evidence that poor maternal diet and excessive GWG predispose offspring to lifelong higher risk of obesity through epigenetic programming [10,11].

Despite these adverse health outcomes, many pregnant women in the United Kingdom and elsewhere fail to adhere to dietary and PA guidelines [12,13], and rates of excessive GWG in many high-income countries are in excess of 50% [14]. Although many countries measure women’s GWG against guidelines, in the United Kingdom, the National Institute for Health and Care Excellence (NICE) guidelines recommend that women not be routinely weighed throughout pregnancy; rather, they should be advised to eat a healthy diet and be physically active and given practical tailored information on how to achieve this [15]. However, reported experiences of women in the United Kingdom and elsewhere suggest that the provision of such prenatal advice is often lacking [16,17]. Health care practitioners cite the lack of time, resources, and confidence, particularly around communicating sensitively and avoiding weight stigma, as well as their own limited knowledge as reasons for not engaging in conversations about diet and PA [18-20].

Numerous interventions have attempted, with varying degrees of success, to encourage healthy eating, PA, and weight management in pregnancy [7]. More recent interventions have used digital technologies either as an exclusive delivery method or to support face-to-face delivery [7,21]. Using an app to deliver such an intervention seems logical, given the widespread use of pregnancy apps today, their broad reach, and their cost-effectiveness [22-24]. Encouraging results from a randomized controlled trial (RCT; n=305) exploring the effectiveness of the HealthyMoms app on GWG, diet, PA, and pregnancy outcomes support the potential of an app-based intervention to promote healthier dietary behaviors and reduce GWG [25].

### Intervention Cocreation

Baby Buddy is a free noncommercial (advertisement free) UK pregnancy and parenting app that guides and supports parents and caregivers throughout pregnancy and their baby’s first year of life, providing them with their own *digital best friend*, interactive features, >300 films, personalized daily information, and signposting to 24/7 support. The app is endorsed by 8 royal colleges and professional bodies, and various aspects of it have been independently evaluated [26,27]. It is integrated into the maternity care pathways of many National Health Service (NHS) trusts across the United Kingdom and, to date, has been used by >350,000 parents. Baby Buddy adheres to the principle of proportionate universalism; although it is available to all expectant and new parents in the United Kingdom, it was created to be particularly relevant to, and engaging for, parents from socially and economically disadvantaged communities whose children are at higher risk of poorer health and developmental outcomes [28]. Originally launched in 2014 by the UK charity Best Beginnings, a new version was launched in 2021 responding to user feedback and findings from a study investigating its use during the COVID-19 pandemic [29]. The updated version includes a dedicated content pathway for fathers and coparents and capacity for new content and functionality that can be unlocked for subsets of app users based on their location, characteristics, or participation in a study or trial. Hence, Baby Buddy presents an ideal vehicle for delivering and evaluating a public health intervention targeting dietary and PA behaviors in pregnancy.

This paper reports on the 3 stages of user research guiding the cocreation of a prenatal healthy eating and PA intervention within Baby Buddy called *Baby Steps to Healthier Habits* (BaSHH) by a team of researchers at University College London (UCL; United Kingdom) and the charity Best Beginnings.

## Methods

### Ethics Approval and Consent

Approval for the research was granted by the UCL research ethics committee (16749/001 and 16749/002). All participants provided informed consent as necessary.

### Intervention Development Process

**Figure 1:**
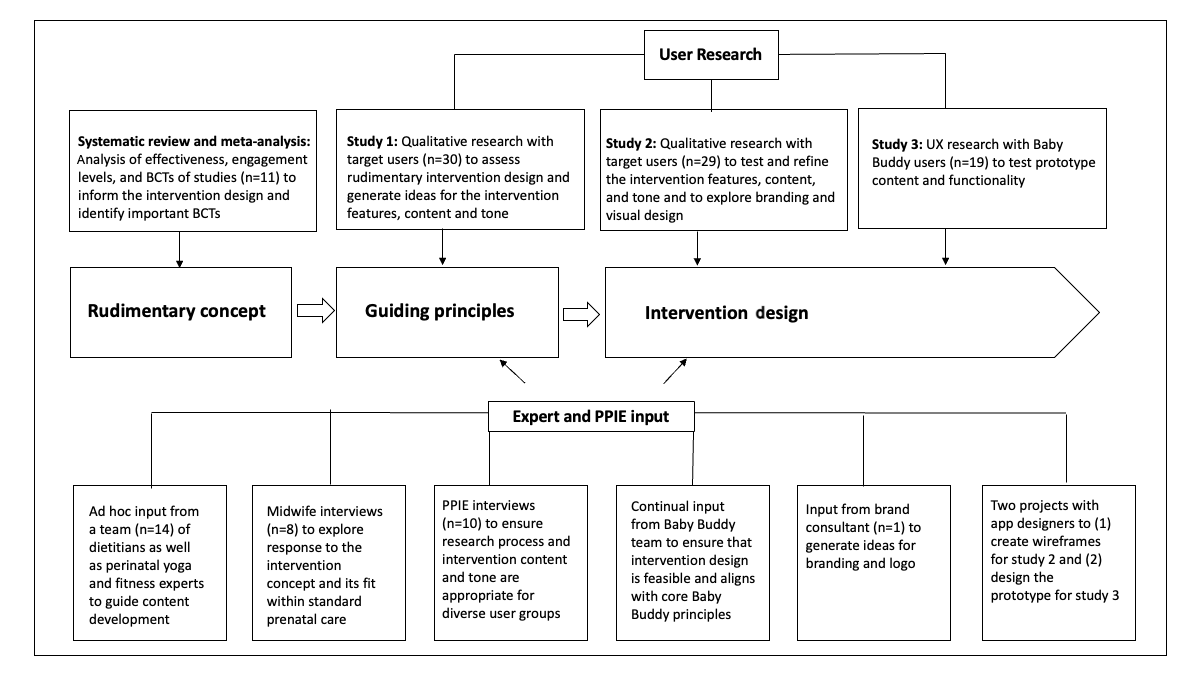
Intervention Development Process. BCT: behavior change technique; PPIE: patient and public involvement and engagement; UX: user experience.

The person-based approach was adopted for the development of this prenatal dietary and PA intervention [30,31]. This method emphasizes the importance of understanding and being guided by the needs and experiences of the intervention’s targeted end users. Two key elements of the person-based approach are the use of qualitative research at every stage of the intervention design process to generate a deep understanding of the end user’s needs and views and the identification of *guiding principles* to inspire and steer the intervention development. Figure 1 illustrates the development process for this intervention.

Three independent qualitative studies were undertaken to capture the needs and wants of the end user. Study 1 assessed reactions to the rudimentary intervention concept, initially described to participants as “A new app feature to help expectant parents develop healthy eating and exercise habits, with information and tips and the ability to set yourselves goals and track your progress,” and explored ideas for its development.

Study 2 explored reactions to the fully developed intervention concept, including the onboarding process; the key goal-setting, self-monitoring, and feedback features; examples of the weekly messages; and branding ideas. Ideas were presented to participants in wireframes and sample copy.

Study 3 tested the user experience (UX) and user interface (UI) using an app prototype to explore reactions to the onboarding and ongoing experience, copy, visuals, layout, flow, and navigation.

A patient and public involvement and engagement (PPIE) group was recruited after the completion of study 1. This group (n=10) comprised individuals from health care and grassroots organizations supporting particular groups and aspects of perinatal health (8/10, 80%) and parents who had used the Baby Buddy app (2/10, 20%). Individual web-based discussions with PPIE group members took place twice between studies 1 and 3, with all participants attending at least 1 session and 6 (60%) of the 10 participants attending both sessions. Additional PPIE interviews were conducted with midwives (n=8) to explore their views of the intervention concept and how they would envisage it fitting into established prenatal care pathways in the United Kingdom.

A wider multidisciplinary team (n=14) that included academic researchers, dietitians, perinatal fitness instructors, and health care professionals contributed to development of the intervention’s evidence-based content by providing guidance on the breadth, accuracy, and tone of the dietary and PA information. In addition, 2 app design consultancies and a branding consultant advised on design of the UI, branding, and logo.

The development of this intervention was guided by the Behavior Change Wheel, at the heart of which lies the capability, opportunity, motivation, and behavior (COM-B) model of behavior change [32], which posits that behavior (B) is part of an interacting system involving a person’s capability (C) to perform the behavior, the opportunity (O) afforded to them to perform the behavior, and their motivation (M) to perform the behavior [33]. For behavior to change, at least 1 of these components needs to be in place or needs to change. The theoretical domains framework (TDF) works alongside the COM-B model, providing an additional layer of granularity by tying the COM-B model to 14 domains of key constructs from established behavior change theories [32]. The intervention has also drawn on elements from the Fogg Behavioral Model (FBM) to direct the process by which users are guided through behavior change and habit formation [34]. The FBM postulates that when a person’s motivation to change is high, challenging behaviors can be undertaken that will help reduce barriers to, and structure, future behavior. However, at times of low motivation, simple and tiny behavior changes provide a more realistic and feasible solution [35].

The rudimentary intervention design was based on the findings of a previously completed systematic review and meta-analysis of exclusively digital health interventions (n=11) targeting diet, PA, and weight gain in pregnant women [36]. The interventions in this review were analyzed and the reported behavior change techniques (BCTs) extracted and coded. The results of the review identified 7 potentially effective BCTs for this target population: goal-setting (behavior), problem-solving, review of behavior goals, feedback on behavior, social support, information about health consequences, and information about emotional consequences. Each of these BCTs was built into the rudimentary intervention design. For social support, a decision was made to test a novel concept of actively including partners in the intervention in a supportive role. This was deemed both congruent with Baby Buddy’s newly embedded pathway for fathers and coparents and feasible, given that all Baby Buddy messaging can be tailored, allowing single women to receive different messages from those with partners. To assess the feasibility and acceptability of such an approach, findings relating to the barriers and facilitators to engaging in this intervention as a couple as well as partners’ roles in pregnancy and their views on healthy eating and PA in pregnancy were investigated and are reported in detail elsewhere [37].

### Study Design

A range of qualitative methods and interview techniques were selected across the 3 studies to best suit the study aims, the sensitivities of the issues covered, and the stimulus materials used. Study 1 comprised a mix of individual interviews and focus groups among female and male participants. Individual interviews were the primary method used to explore female participants’ views of how individual pregnancy experience might affect response to the intervention. For male participants, focus groups were chosen because the camaraderie of the group setting was deemed to be more comfortable for male participants, given their likely lack of familiarity with discussing lifestyle behaviors in pregnancy. The COVID-19 pandemic necessitated a deviation from the original study protocol of face-to-face interviews to remote interviews. The focus groups were conducted on a videoconferencing platform and the individual interviews by telephone. An a priori decision was made to recruit a sample size of 15 male and 15 female participants.

Study 2 was designed from the outset as web-based research. Individual interviews with female participants and couples were conducted on a videoconferencing platform.

Topic guides for studies 1 and 2 were designed using open-ended questions. Discussions were largely participant led, allowing participants to comment on, and generate ideas for, the aspects of the intervention of most interest and concern to them, although the moderators (AR and AP) ensured that the core topic areas were covered in each session.

**Figure 2:**
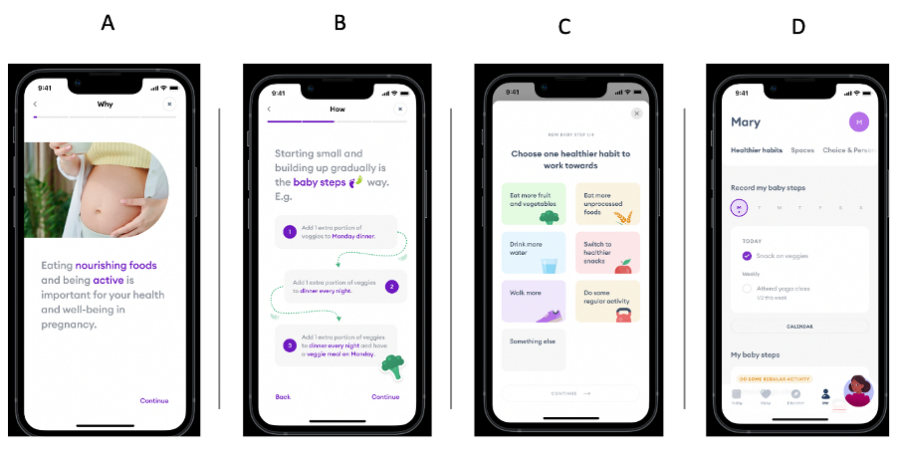
Examples of prototype screens. (A) Example of an onboarding screen, providing evidence-based information on the importance of healthy eating and physical activity in pregnancy. (B) Screen illustrating the concept of baby steps building toward healthier habits. (C) First screen in the process of setting up a healthier habit to work on. (D) Example of a user’s dashboard where they can record a baby step.

Study 3 investigated the UX and UI through a series of web-based *think-aloud* interviews. This interviewing technique encourages participants to engage with an intervention, saying aloud any thoughts that come into their heads as they work through it. In this instance, participants were asked to use the app prototype as they would in real life, voicing their thoughts as they did so. With the participant sharing their screen, the researcher was able to follow the sequence, noting the speed and ease of navigation through the digital prototype. The researchers interjected if participants were experiencing problems but otherwise waited until the end of the screen sequence to ask additional questions. Figure 2 illustrates some example screens from the app prototype.

Sample sizes for studies 2 and 3 were determined by the data saturation method: once no new data and insight were being uncovered in the interviews, the interview process was deemed completed [38].

### Participants

Participants for studies 1 and 2 were either expectant parents in the last trimester of pregnancy or parents of a baby (aged <18 months). Consistent with Best Beginnings’ policy of addressing health inequalities, the primary recruitment focus was on participants from economically or socially disadvantaged communities, including those without tertiary education, on low incomes, and from minority ethnic groups. In study 2, additional screening questions were introduced to exclude those who scored themselves as ≥7 for healthy eating and keeping fit, where 10 was excellent and 0 very poor. This ensured that the sample comprised those who would benefit most from the intervention rather than those already predisposed to healthier habits. Study-3 participants were a self-selecting sample of current Baby Buddy app users who responded to an in-app push notification asking for participants to test a new feature.

### Recruitment Procedure

Recognizing the challenge of recruiting participants from minority ethnic groups and low-income groups to take part in a qualitative research study, an a priori decision was made to use professional market research recruitment services to assist with recruitment for studies 1 and 2. Additional study-1 participants were recruited from a convenience sample of those who had previously completed a national web-based survey conducted by Best Beginnings, Home-Start UK, and Parent-Infant Foundation. This web-based survey, conducted between April 2020 and June 2020, explored the views and experiences of expectant parents and parents of very young babies during the COVID-19 pandemic [39]. At the end of the survey, participants indicated whether they would be willing to take part in further research. Additional study-2 participants were recruited through social media. In both studies, participants received an incentive of £25 (US $32.5) per individual or £40 (US $52) per couple. Study-3 participants were recruited via an in-app notification asking for volunteers to participate in a web-based interview with a UCL researcher to test a new Baby Buddy feature.

### Data Collection

Study-1 focus groups and telephone interviews were moderated by AR, an experienced qualitative researcher, between July 2020 and September 2020. AR and AP conducted study-2 interviews between November 2021 and April 2022 and study-3 interviews between May 2022 and June 2022.

### Data Analysis

Study 1 applied inductive thematic analysis to analyze the data [40]. All focus group and telephone interview audio recordings were transcribed and uploaded to NVivo 12 Pro (QSR International) [41]. AR and AS developed an initial coding structure and independently coded 50% of the data. Any discrepancies between AR and AS regarding codes or coding definitions were resolved through discussion. AR coded the remaining 50% of the transcripts. AR and AS identified themes through an iterative process of discussion, refinement, and development. AR revisited the transcripts to confirm the legitimacy of the final themes.

Study 2 adopted an iterative approach to data analysis with feedback from early interviews resulting in changes to the intervention that were tested out in later interviews. Consistent with the person-based approach, a *table of changes* [42] was used to systematically record proposed and actual changes to the intervention delivery and content. All positive and negative comments that might affect the intervention design were logged, and corresponding suggestions for changes were discussed and agreed on by AR and AP in conjunction with the Baby Buddy team. The MoSCoW (Must Have, Should Have, Could Have, and Will Not Have this time) prioritization framework was used to rank the importance of the proposed changes.

Study 3 was also an iterative cycle of user feedback and design modifications. Discussions took place with the Baby Buddy team and app designers after each 3 to 4 interviews, with resulting changes to the prototype screens being made throughout the course of the fieldwork.

## Results

### Overview

A total of 30 participants took part in study 1. Three focus groups were conducted among the male participants (15/30, 50%). Of the 15 female participants , 12 (80%) took part in one-on-one telephone interviews, and 3 (20%) participated in a mini focus group. The focus groups lasted up to 90 minutes, and the interviews lasted between 45 and 60 minutes.

A total of 29 participants took part in study 2. Of the 29 participants, 15 (52%) female participants took part in individual interviews, whereas the remaining 14 (48%) participants (7 couples) participated in couple interviews. The interviews were between 40 and 60 minutes long.

A total of 19 participants (n=18, 95% female and n=1, 5% male) took part in study 3. All interviews were conducted with female participants and lasted 30 to 45 minutes.

### Participant Characteristics

Participant characteristics for each study are summarized in Table 1. Study-1 participants were aged between 18 and 44 years. More than half the sample (16/30, 53%) were of non-White ethnicity, and nearly three-quarters (22/30, 73%) had no education beyond secondary school, including 18% (4/22) who had no educational qualifications. The majority of the participants were first-time parents with a baby aged <12 months (23/30, 76%). Study-2 participants were aged between 22 and 39 years. Three-quarters of the sample (22/29, 76%) were of non-White ethnicity, and 45% (13/29) had no education beyond secondary school. The study-3 sample was older, with 42% (8/19) of the participants being aged >36 years, and educated to a higher level, with 84% (16/19) being graduates and postgraduates. All samples were geographically spread across the United Kingdom (excluding Northern Ireland).

**Table 1:** Characteristics of all study participants (N=78).

			Study 1 (n=30), n (%)	Study 2 (n=29), n (%)	Study 3 (n=19), n (%)
**Sex**					
		Female	15 (50)	21 (72)	18 (95)
		Male	15 (50)	8 (28)	1 (5)
**Age (years)**					
		<20	1 (3)	0 (0)	0 (0)
		20-29	7 (23)	13 (45)	4 (21)
		30-39	19 (63)	16 (55)	7^a^ (37)
		≥40	3 (10)	0 (0)	8^b^ (42)
**Ethnicity**					
		White	14 (47)	7 (25)	11 (58)
		Black	8 (27)	10 (34)	3 (16)
		Asian	7 (23)	9 (31)	4 (21)
		Mixed	1 (3)	3 (10)	1 (5)
**Education**					
		None	4 (13)	1 (3)	0 (0)
		GCSE^c^	12 (40)	4 (14)	1 (5)
		A levels	6 (20)	8 (28)	2 (11)
		Graduate	5 (17)	10 (34)	7 (37)
		Postgraduate	2 (7)	3 (10)	9 (47)
		Not stated	1 (3)	3 (10)	0 (0)
**Pregnancy or baby status**					
		First pregnancy or baby	25 (83)	18 (62)	16 (84)
		Second or higher pregnancy or baby	5 (17)	11 (38)	3 (16)
		Pregnant	2 (7)	8 (28)	11 (58)
		Baby aged <6 months	13 (43)	4 (14)	0 (0)
	Baby aged 6 to 12 months		10 (33)	14 (48)	8^d^ (42)
	Baby aged 13 to 18 months		5 (17)	3 (10)	0 (0)

^a^Aged 31 to 36 years.

^b^Aged >36 years.

^c^GCSE: General Certificate of Secondary Education.

^d^Baby aged <18 months.

### Study 1

#### Overview

Participants responded positively to the rudimentary intervention concept of an app feature to support expectant parents to develop healthier dietary and PA habits. Four main themes were identified relating to the barriers and facilitators to engaging in the intervention as a couple, which are reported elsewhere [37]. Eleven themes were identified with respect to expectations and desires for the intervention, 3 of which related to the intervention content and 8 to intervention style (Textbox 1).

Responses were broadly consistent across the sample, with minor differences reported in the following sections.

**Themes from study 1.**

**Intervention content**

- Providing a “Why?”
- Explaining the “What?”
- Providing guidance on the “How?”

**Intervention style**

- Support me
- Make it easy to use
- Make it fun to use
- Keep us interested
- Give us flexibility
- Make it shareable
- Reward us
- Make it personal

#### Theme 1 (Content): Providing a “Why?”

Participants were unanimous in their opinion that rather than simply instructing participants to eat a healthy diet, keep physically active, and achieve a healthy weight gain, this intervention should emphasize and explain why these behaviors and goals are so important during pregnancy and to future family life. Many of the participants felt that the ubiquity of healthy lifestyle messages had diluted their potency to the extent that they had become easy to ignore. Accordingly, being explicit about the benefits to maternal and fetal health was considered essential, both to strengthen the rationale for engaging in the intervention and to fill gaps in users’ knowledge:

If it says this is harmful to your baby or this will be more beneficial instead. I think that would probably work better than...I mean all the medical professionals, the dietitian and everyone’s all banging on about how good fruit and veg. and everything is.

Male participant #1

I know young mums and all that are actually really curious about the diets and all that...When I was pregnant, I had a lot of questions, but I forgot what they were, but I remember some of them weren’t answered, but a place where you could get them answered would be really good.

Female participant #2

Although learning about the risks associated with poor diet and an inactive lifestyle during pregnancy was considered important, the issue of excessive GWG provoked a divisive response. Some of the female participants, particularly those who had experienced postpartum weight retention, were keen that the intervention should communicate the risks of excessive GWG. However, others were adamant that, because it is a highly emotive issue, the topic of GWG should be avoided. Participants with overweight or obesity were particularly vociferous about their dislike of focusing on weight gain, reporting that it would induce feelings of guilt at a time when weight gain was inevitable and as such was likely to prompt their disengagement with the feature. Moreover, most of the female participants believed that the level of weight gain in pregnancy was idiosyncratic; they were guarded and doubtful regarding the idea of a correct amount:

Speaking as someone who’s had weight problems most of my life, I'm trying to have a mindset of I want to be healthy...getting “well done” (on your weight) is probably not so good.

Female participant #5

#### Theme 2 (Content): Explaining the “What?”

Participants were keen for the intervention to provide comprehensive advice on what exactly constitutes healthy eating in pregnancy, beyond the generic messages such as *5 portions of fruit and vegetables a day*. Many of the participants felt that this advice had been missing in their prenatal care. Few participants were interested in understanding dietary requirements at a nutrient level, with most simply wanting advice on what foods to eat to ensure that their baby got the nutrients it needed. In addition, participants were keen for the intervention to address misinformation and myths about diet in pregnancy, in particular the idea that pregnant individuals should be “eating for two”:

If someone was to tell him [partner] maybe don’t have too much sugar during your pregnancy...because they don’t know. All they hear from their mothers is eat for two. They probably think they are doing the right thing by feeding me.

Female participant #15

Guidance on PA was seen to be equally important. Participants, male participants especially, were often unsure as to what amount or type of PA was safe during pregnancy, leading to an assumption that avoidance of all but walking was the safest option:

I think just some information on kind of what you can actually do, ’cause I think I was just probably playing it safe with walking. That’s pretty boring. There might be other things that you can do that you just don’t really know about.

Female participant #3

#### Theme 3 (Content): Providing Guidance on the “How?”

Participants were keen for the intervention to show them how to develop healthier dietary and PA habits by giving them both practical advice and tools for the behavior change process, as well as strategies for avoiding the common pitfalls. There was considerably less interest in the provision of information about, or functionality for, weight monitoring for reasons discussed in the *Theme 1 (Content): Providing a “Why?”* section. Although this research did not explore barriers to healthier energy balance behaviors, participants were keen for the intervention to include topics such as pressure from friends and family; eating healthily on a budget; finding the time, energy, and space for PA after a hard day at work; or coping with the pressures of childcare:

Ways that you could easily fit it in during the day. You could do this particular thing while you’re waiting for the kettle to boil.

Female participant #8

Participants identified the goal-setting feature and the ability to self-monitor or track behaviors as being important tools to help them develop healthier habits. Reminder notifications to complete or record a behavior were regarded as essential, and some of the participants suggested a diary to record more qualitative elements of their journey.

In terms of feedback on their progress, participants desired weekly personalized notifications summarizing their achievements and providing appropriate congratulations, encouragement, or tips on how to improve performance. In addition, notifications when goals were achieved were suggested by some of the participants:

Feedback always helps—it’s really good because then it’s not a one-way thing. If you are getting something back it definitely drives you on. And there’s that competitive element as well—that reward. You want to do better and achieve more...Competitive against yourself

Female participant #6

Participants wanted the intervention to include an extensive range of healthy eating and PA ideas. They were particularly keen on recipe ideas that were quick, easy, cheap, and achievable for those with limited cooking experience:

Having a recipe resource is obviously very useful, especially if it's going to be food that is gonna be beneficial to the baby as well.

Male participant #6

So, I think if there’s like quick, convenient, kind of like meals on there and things and maybe like tips and things to get more protein and things like that into diet without being in the kitchen for hours and hours.

Female participant #3

The participants also stated that the recipes should ideally cover world cuisines and include vegetarian and vegan options. Several of the participants suggested a feature enabling users to share recipe ideas with other users. Similarly, PA ideas were expected to offer a wide variety of recommendations, including short, simple exercises that can be performed at home, as well as longer workout routines.

Male participants wanted advice and guidance on how they can provide practical and emotional support to their partners in achieving healthy eating and PA goals:

What I would suggest putting into the app would be practical things that the man can get more involved with...for example, there was some meals that could be cooked easily by a man, which would benefit the pregnancy development.

Male participant #10

#### Theme 4 (Style): Support Me

Participants asserted that, although the intervention should be informative, it should avoid an authoritative or instructive tone. They did not want to be told what to do, to be patronized, or made to feel guilty in any way. Rather, they felt that the intervention should offer support and encouragement while acknowledging the many challenges facing pregnant women:

But it’s about delivery, you don’t want it to be condescending—the midwife making you feel guilty. You need to make sure there is a certain level of equality in how you speak to parents, then you won’t get men protesting or anything.

Male participant #7

Sometimes around pregnancy things can come across as a bit naggy, but useful tips that you can either take on board or not is a good idea.

Female participant #10

#### Theme 5 (Style): Make It Easy to Use

Of paramount importance was that the intervention and all its features should be easy to use. Many of the participants talked about downloading apps that they subsequently failed to use because of the effort they required either to set up or to use once set up. In particular, if setting goals and recording behaviors were not intuitive or required too much effort, participants felt that they would be unlikely to use such functionalities. Information was expected to be easily accessible and delivered in bite-size chunks typical of social media apps. The use of video and visual images was suggested to reduce reliance on text, with links to more detailed articles for those interested in reading more on a particular topic:

Whenever I have used an app, it depends on how easy it is. Some you have to try hard to find the information—if they are accessible and easy it would be more appealing.

Male participant #8

I’d like something that was continuously reminding you, like small notifications—like an Instagram pop-up—maybe it’s just my age group but that would help me focus more rather than a message that says there’s a new blog out, read it.

Female participant #15

#### Theme 6 (Style): Make It Fun to Use

Although participants appreciated that the subject matter was serious, they were adamant that the intervention should be fun to use. In this respect, they suggested the inclusion of unusual and interesting facts, as well as fun challenges:

And maybe like a lot of fun facts like, the Romans used to eat this kind of food or how it was made. I think funny things as well—make it not sound really, really serious.

Female participant #2

Interesting graphics to illustrate an individual’s progress toward a goal and token rewards were also thought to be important:

I had a Fitbit before I had the baby and I think that worked really well. You set the step count you want to achieve in a day and when you hit that it would flash and the whole screen would be taken over with rainbows and fireworks.

Female participant #6

#### Theme 7 (Style): Keep Us Interested

Growing bored with an app, particularly fitness tracking apps and other apps where the content remained the same over time, was something that many of the participants saw as a barrier to use. Although regular notifications of new content were seen as the most obvious way of averting boredom, other suggestions included tying the program to the developmental stages of the baby to create a sense of change and progression, introducing regular new challenges, and encouraging new goal-setting at various stages. Regular personal feedback, sharing posts with other participants, and rewards were also suggested as ways of maintaining interest.

#### Theme 8: Give Us Flexibility (Style)

Participants were keen for the intervention to be tailored to meet an individual’s needs as much as possible. Flexibility was sought in terms of how they could engage with the intervention (eg, giving participants the option to amend the intervention to focus solely on diet, PA, or both and to choose whether to participate with or without a partner). Flexibility around goal-setting was also considered essential to allow individuals to choose their preferred type and frequency of the behavior. Participants wanted to be able to set the timing and frequency of reminders to complete the behavior and record it. The ability to opt out of reminders was also thought to be important.

#### Theme 9 (Style): Make It Shareable

Participants expected an intervention targeting both parents to include dedicated spaces within the app for couples to share their progress and highlight information, tips, and challenges of particular interest. Presenting the use of such spaces as optional was deemed important, given that some users might want to use the feature on their own. Many of the participants, including single parents, were keen to allow for flexibility to extend the concept of shared spaces to other family members or friends or even a prenatal group to increase motivation, inspire each other, and share ideas and tips:

Could there be an area like a forum on the app where you could discuss healthy eating options? Having peer support was really important for me.

Female participant #4

Views on whether progress data should be shareable were mixed. Although some male participants thought the idea of competition among intervention participants was motivating, others, especially female participants, felt that it may cause pressure and, as such, was inappropriate for a pregnancy app.

#### Theme 10 (Style): Reward Us

All participants felt that token rewards in the form of fun graphics illustrating an individual’s progress were important. A small minority, particularly male participants, suggested that rewards should be tangible in the real world in the form of vouchers toward baby equipment or discounts on life insurance policies.

#### Theme 11 (Style): Make It Personal

Participants were very keen for the user experience to be as tailored and personal as possible, particularly with respect to feedback. Messages of congratulations, support, and motivation were expected to be tailored according to an individual’s progress. Using a participant’s name in messages was generally approved of:

I think you need that feedback, I think, either a congratulatory sort of feedback is great because you’ve done it really, really well...[or] you’ve done it eighty percent of the time or whatever, here are some ways that you could perhaps make it a bit easier...those extra little tips and pointers would be great.

Female participant #8

#### Mapping Themes to the COM-B Model and the TDF

A summary of the themes as mapped to the COM-B model and the TDF can be found in Multimedia Appendix 1. Initial ideas that were generated for intervention function and BCTs are also included.

### Guiding Principles and Intervention Concept Development

On the basis of the findings from study 1, the guiding principles underlying the intervention were developed and agreed with the wider Best Beginning team as follows:

To empower expectant parents together to replace unhealthy diet and PA habits with healthier alternatives, through building knowledge, skills, and confidence in an easy and enjoyable way.

With input from experts in various fields (dietitians and nutritionists, perinatal personal trainers, community health program leads, psychotherapists, an eating disorders specialist, and app designers), the intervention was built around the key participative features of goal-setting, self-monitoring, and feedback, with the COM-B analysis used to generate ideas for other relevant BCTs. An onboarding script was written to introduce new users to the intervention and guide them through the process of setting up their healthier habit goals and the first behavior change steps. To support this, a 32-week program of gestational age–appropriate bite-size messages covering dietary and PA information, fun facts, tips, recipe ideas, and challenges was created. Examples of these messages can be found in Multimedia Appendix 2.

### Study 2

This iterative research study with 29 participants generated a series of amendments and refinements to the intervention concept, onboarding script, branding, and weekly messages, examples of which are shown in the *table of changes* extract (Table 2).

Key learnings from this research confirmed the initial stage 1 findings that participants, especially those with obesity or experience of an eating disorder or disordered eating, disliked the idea of the intervention including GWG monitoring and focusing too much on weight messages. Indeed, many of the participants felt that this would be a barrier to engagement:

They don’t weigh you now as it’s detrimental to mental health. You don’t want any pressure or stigmatism [stigma] about gaining or losing weight.

Female participant #10

The reality is a lot of women put on weight during pregnancy. Our bodies are not all the same...you need to know but if you’re made to feel guilty, I think you’d just give up.

Female participant #2

Focusing on diet quality and PA was considered substantially more motivating and pertinent in that most of the participants were more familiar with the notion of a link between maternal diet and fetal health than one between GWG and fetal health:

I like food and I like unhealthy food...but if it said “be your best for your baby”...

Female participant #4

This research also provided essential insight into terminology and tone:

If you say “mums” it’s like you’re coming alongside us. If you say “mothers” it’s like you’re telling us to do something.

Female participant #11

An important learning was that *healthy eating* was not always considered to be an appealing or motivating term to describe the concept because of its ubiquity and its association with expensive ingredients:

Healthy eating you think of expensive things—like organic...smoothies and salads with exotic vegetables.

Female participant #1

Although *healthy* or *healthier* was considered acceptable in the context of choices and swaps, *nutritious foods* or *foods full of important nutrients* seemed to be more motivating and reinforced the notion of a pregnancy and fetal health–specific intervention.

This stage of research also reinforced the importance of personalization in terms of both being able to choose your healthier habits and baby steps and feedback on progress. Flexibility was regarded as especially important during pregnancy, given female participants’ wide range of experiences and challenges:

It’s adapted for me—where I have done well and where I can improve. I couldn’t be as active as I wanted to. I had to have a lot of bed rest, but with this it wouldn’t matter.

Female participant #8

It didn’t say “you must do this”—it was “what sort of goals do you want to set,” which is so much better. I got gestational diabetes diagnosed at 8 weeks and I ended up with pelvic girdle pain, but I still would have done this.

Female participant #10

**Table 2:** Extract from the table of changes.

Content and positive comments		Negative comments	Possible change	Reason for change	MoSCoW^a^
**Onboarding**					
	Recognizes the challenges facing pregnant women: “I like that it understands—you can get all hormonal and feel like an absolute failure.” [Female participant #11]	Feels aimed at first-time parents: “I’ve had a child before, but I still ask these questions.” [Female participant #2]	Include more references to additional challenges of having other children	IMP^b^	Must do
	Explains why healthy eating and PA^c^ are important now to mother and baby: “We’ve heard the general stuff since we were children and haven’t done it—the only way we will now is if it’s for baby.” [Female participant #11]	Distal health benefits are not especially motivating: “We are all going to die of something—I want to know about feeling good now—good for your energy, mentally brighter.” [Female participant #4]	Focus on proximate benefits to mother’s and baby’s health	IMP	Must do
**Weekly messages**					
	Bite-size messages are good: “I like little notifications that pop up on my phone during the day.” [Female participant #5]	Some messages are too long: “I get bored of reading.” [Female participant #5]	Cut down longer message even further	REP^d^	Must do
**Dietary message**					
	Relevant to most	Not always appropriate for women with GDM^e^	Consider additional messaging for women with GDM	NCON^f^	Could do
**Self-monitoring**					
	Good to have reminders	“There should be a snooze option there—remind me in an hour.” [Female participant #2]	Would like a “remind me later” option	NCON	Should do
**Rewards**					
	Nice to have achievement recognized: “Even nice emojis—just makes you feel you’ve achieved something.” [Female participant #5]	Token rewards have no real value	Would like real-value rewards such as discounts on healthy foods	NCON	Would like
**Branding**					
	Baby Steps fits with Baby Buddy and communicates the method; little feet logo appeals: “The name [Baby Steps] makes it feel not so daunting.” [Female participant #4]	Other brand names/logos have limited appeal: “Not Healthy Homes—it sounds very NHS [National Health Service].” [Male participant #7]	Go with Baby Steps	REP	Should do

^a^MoSCoW: Must do, Should do, Could do, and Would like.

^b^IMP: important for behavior change or guiding principles.

^c^PA: physical activity.

^d^REP: repeatedly mentioned in research.

^e^GDM: gestational diabetes mellitus.

^f^NCON: does not contradict guiding principles.

### Final Intervention Design

**Figure 3:**
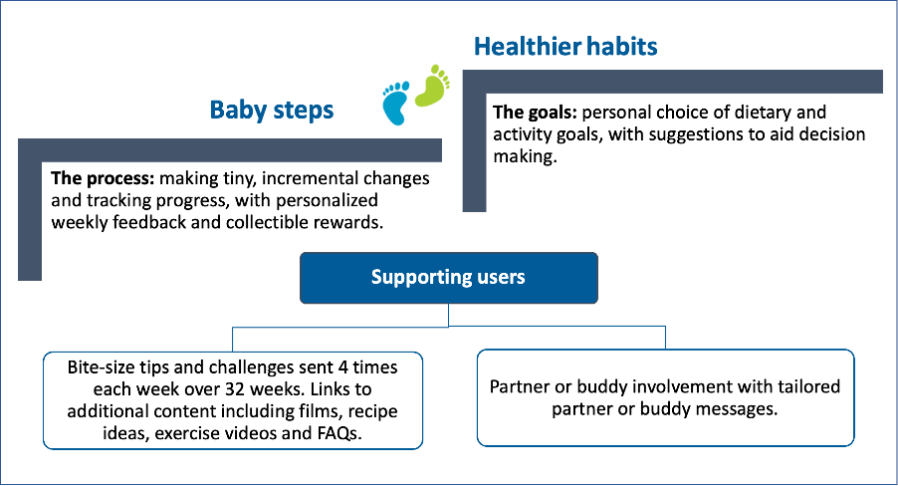
Final intervention design. FAQ: frequently asked question.

Figure 3 provides an overview of the intervention design and branding emerging from studies 1 and 2 as well as the PPIE input. Examples of the messages sent to users can be found in Multimedia Appendix 2.

### Study 3

A similarly iterative process to study 3 (n=19) led to several changes to the UX and UI. The most significant UX change was shortening of the onboarding process. Participants felt that although the information was interesting and important, the current length might act as a barrier to completing the onboarding process:

It’s too long but it’s good for getting women thinking about healthier habits...it takes too long to get to actually setting up your first baby step.

Female participant #6

A subsequent iteration of the prototype shortened the onboarding process by providing a list of suggested healthier habits and relocating some of the content to follow-up push notifications:

I like that you can click on one of the habits. It would be even better if there was a drop-down menu of suggested baby steps, so you didn’t have to stop and think of one there and then.

Female participant #7

Watching and listening to participants navigate through, and respond to, the screens during the *think-aloud* interviews helped to identify the messages that were particularly engaging and motivating, as highlighted by a participant in this observation:

Saying how it’s important for your baby and having the scan picture is really good—that would have made me want to do it—you’re doing it for your baby.

Female participant #13

The process also exposed potential areas of confusion with terminology and navigation:

It’s confusing having a calendar and a diary...maybe call it a journal or my progress [instead of diary].

Female participant #10

These interviews provided valuable feedback on the UI, confirming the appeal of background visuals, emojis, and highlighted text and indicating where UI improvements needed to be made:

I like the purple words—it draws you to what’s important if you’re reading through quickly.

Female participant #11

Participants also shared their views on the proposed reward scheme. Although the collectible icons or badges relating to the different healthier habits had broad appeal, several of the participants noted that the egg icon would not appeal to those on a vegan diet:

I really like that you can see all the awards, then you know what you are reaching for...you get drawn in like “I want that one.”

Female participant #10

Finally, the responses to the prototype provided insight into how to use branding and visual imagery to strengthen and clarify communication of the concept of taking baby steps toward a healthier habit goal:

To me those little feet say baby steps—like a shorthand.

Female participant #9

The feedback from this study was used to make changes iteratively to the prototype until the app designers, the UCL team, and the Baby Buddy team agreed on the final design to be built.

## Discussion

### Principal Findings

This 3-stage user research study to develop an app-based digital intervention promoting healthy eating and PA for expectant parents used the person-based approach to ensure that the needs and opinions of target users were understood and accommodated at each stage of the development process. Cocreation with the parenting charity Best Beginnings combined the strengths of academic rigor with real-world experience of designing effective digital health products. The overarching intervention structure was strongly theory based, drawing on the COM-B model to identify potentially effective BCTs and on the FBM to guide the design and process of user engagement with key BCTs [34,43]. User feedback at each stage (n=78 total participants) of the intervention development process shaped the content, functionality, style, and tone of the intervention.

### Comparison With Existing Literature

To our knowledge, this is the first prenatal dietary and PA intervention to have been developed using the person-based approach. Adopting this approach resulted in an intervention design that differs substantially from previous prenatal dietary and PA interventions in that it targets expectant couples rather than pregnant women alone, and it excludes weight monitoring as well as weight messaging. The premise for designing a couples’ intervention is reported elsewhere [37]. The decision to omit a weight monitoring feature and focus on healthy eating and PA rather than GWG-related messaging was based on the comments by participants, in particular those who had experienced difficulty with their weight, that weight messages were demotivating and a potential barrier to engagement. This approach contrasts with that of other existing digital interventions that aim to promote healthy eating and PA and prevent excessive GWG in pregnancy [25,44,45]. The extent to which weight monitoring contributes to the effectiveness of multifaceted digital interventions is unclear. The use frequency of weight monitoring features is rarely reported, but in 1 large RCT (n=1689), although 70% of the participants used the feature once, continued use over an average 28-week period was very low (median use 3, IQR 0-9 times) [46]. Research has reported on pregnant women’s discomfort discussing weight issues and dislike of regular weight monitoring [47,48]. Moreover, intervention trials in the United Kingdom have reported no effect of routine prenatal weighing or providing women with GWG guidelines [49,50]. Accordingly, sidestepping weight messaging and focusing instead on the more widely motivating message of eating nutritious foods and keeping active to promote maternal and fetal health might be a more effective way of reaching those at greatest risk of excessive GWG. This also avoids risk of unintentionally triggering weight-related anxiety, especially in women with a history of disordered eating or eating disorders for whom bodily changes during pregnancy can be distressing [51].

Unlike many other countries, the United Kingdom does not use GWG guidelines. Rather, women are advised to eat a healthy diet and be physically active during pregnancy [15]. However, consistent with previous research, this study indicated that, currently, advice is often limited and not necessarily tailored to individual circumstances [17]. Again, consistent with other research, this study’s PPIE interviews with midwives indicated that the lack of time, confidence, skills, and expertise prevented them from delivering comprehensive and tailored advice on healthy eating and PA [52]. As such, this self-contained intervention could serve to support midwifery services and alleviate pressures on scarce health care resources.

The level of attrition—defined as dropping out or engaging minimally—in digital healthy lifestyle interventions is typically high, especially in longer-term interventions [53-55]. Few intervention studies report on reasons for dropping out. However, those that have, as well as studies investigating why users abandon commercial health apps, suggest several causes, including boredom, the loss of novelty, the lack of time, high data entry burden, the loss of motivation, and the lack of personalized feedback [56,57]. These reasons for attrition are echoed in the themes identified in study 2, highlighting the importance of regular and varied intervention content to hold users’ interest. In particular, to minimize user burden with regard to the goal-setting and self-monitoring features, simplicity and ease of use were identified as pertinent. Personalized feedback was mentioned as an aspect of a digital lifestyle intervention to continue to motivate users. Similar themes have been reported in a previous systematic review of digital services (n=23) promoting PA and healthy diet in the context of noncommunicable disease prevention [58].

The feedback in study 3 highlighted the importance of a swift and easy onboarding procedure. This resulted in some unavoidable deviations from the planned design of the BCTs and raises the question of the transferability of face-to-face BCTs to self-directed digital formats. Previous studies have shown how the delivery method can affect intervention effectiveness and the role of BCTs [44,59]. This is especially true as health care systems are stretched and looking to scalable digital mobile health (mHealth) programs to alleviate some of these ongoing pressures.

Further research is needed to understand the impact of delivery format on BCTs, and the recently developed mode of delivery ontology will facilitate this [60]. Gamification (using elements of game playing in nongame contexts) has been suggested as a way of optimizing engagement with digital health behavior change interventions [61]. Applying the person-based approach to this research allowed target users to generate and refine ideas for appealing gamification strategies, including the addition of shared space for the exchange of ideas among partners, family members, or friends and a token reward scheme for the duration of the intervention to maintain engagement.

### Future Directions

As a feature within an existing free app that is already integrated into NHS maternity care pathways in the United Kingdom, BaSHH has the potential to be delivered at a national level at no extra cost to health care providers already using Baby Buddy in their maternity care pathway. The next important step is to test its effectiveness in improving dietary and PA behaviors and reducing rates of excessive GWG. Initially, BaSHH will be added to Baby Buddy in up to 3 locations within the United Kingdom so that researchers can undertake a service evaluation and feasibility study to determine its acceptability and provide preliminary feedback on its effect on dietary and PA behaviors and GWG. If appropriate, an RCT will then test its effectiveness in improving dietary and PA behaviors and reducing rates of excessive GWG.

Designing BaSHH has enabled us to create a framework and add new goal-setting, self-monitoring, and feedback functionalities to Baby Buddy that can be used to build further behavior change features supporting expectant and new parents in smoking and alcohol cessation, perinatal mental health, postpartum weight loss, and family health behaviors. Further collaborations between academics and Best Beginnings are envisaged to realize Baby Buddy’s potential as a national digital health intervention.

### Strengths and Limitations

A strength of this intervention development was that it combined a theory-based approach with adherence to the principles of the person-based approach. Although behavioral theory informed the main constructs of the intervention, continual feedback from target users ensured that their needs and opinions shaped the design and content. Loosely structured topic guides, the use of open-ended questions, and *think-aloud* sessions allowed participants to explore and discuss elements of the intervention that interested them and to generate their own ideas for intervention content, style, and tone. Importantly, this approach also helped to mitigate unintended consequences, such as deterring potential users by focusing on weight messages. In addition, establishing and adhering to a set of *guiding principles* ensured that all those contributing to the intervention design were working toward a common vision. A further strength of this research was that, consistent with Best Beginnings’ commitment to taking a proportionate universalism approach to improve outcomes and reduce inequalities, the sample represented those from economically and socially disadvantaged population groups who are the most at risk of poor dietary and PA behaviors and excessive GWG. However, a limitation of the research was the potential for self-selection bias in the sample. Participants in each stage of the research were preinformed about the topic of discussion. As such, there was a risk that participants were more open to the concept of a healthy eating and PA intervention than a truly random sample might have been. We attempted to mitigate this in study 2 by screening out potential participants who rated themselves highly on healthy eating and keeping fit. In addition, the participants all had internet access, and many of them were current users of pregnancy apps.

A further limitation of this study is that it took place during the height of the COVID-19 pandemic. This meant that, contrary to the original study design, none of the planned interviews and focus groups were conducted face to face, potentially compromising the richness of the data. Study 1 had been due to start in March 2020, just as the first COVID-19 national lockdown started in the United Kingdom. The commencement of the study was delayed to avoid the effects of stay-at-home orders influencing the robustness of research findings. An interim study exploring the effects of COVID-19 on Baby Buddy users highlighted COVID-19–related changes in dietary and PA behaviors and app use [29]. The key findings from this study alerted the project team to the important impact of COVID-19 on the overall study design and findings.

A final limitation of qualitative research methods more generally is the influence of the researcher in both moderating the sessions and interpreting the findings. Nevertheless, across the 3 presented studies, a team of 4 researchers ensured a rigorous data collection and analysis process to minimize researcher bias and deliver valid and reliable findings.

### Conclusions

This study describes the cocreation and development process of an app-based intervention to support healthier dietary and PA behaviors and weight management in pregnancy. It illustrates the value of combining a theoretical method for intervention development with the person-based approach to create a theory-based intervention that is also user-friendly, appealing, and engaging for its target audience. Further research is needed to evaluate the effectiveness of the intervention in improving dietary and PA behaviors and reducing rates of excessive GWG.

## Appendix

Multimedia Appendix 1 Themes from study 1, as mapped to the capability, opportunity, motivation, and behavior (COM-B) model and the theoretical domains framework (TDF).

Multimedia Appendix 2 Examples of bite-size messages.

## Abbreviations

BaSHH: Baby Steps to Healthier Habits
BCT: behavior change technique
COM-B: capability, opportunity, motivation, and behavior
FBM: Fogg Behavior Model
GWG: gestational weight gain
mHealth: mobile health
MoSCoW: Must Have, Should Have, Could Have, and Will Not Have this time
NHS: National Health Service
NICE: National Institute for Health and Care Excellence
PA: physical activity
PPIE: patient and public involvement and engagement
RCT: randomized controlled trial
TDF: theoretical domains framework
UCL: University College London
UI: user interface
UX: user experience

## Data Availability

The interview transcripts generated and analyzed during this study are available from the corresponding author on reasonable request.
