# Supplementary figures and images for: Using the Person-Based Approach to Develop a Digital Intervention Targeting Diet and Physical Activity in Pregnancy: Development Study
Source: JMIR Form Res. 2023 May 26;7:e44082. doi: 10.2196/44082 (PMC10257111; doi:10.2196/44082)

**Multimedia Appendix 1.** Themes from Study 1, as mapped to COM-B.


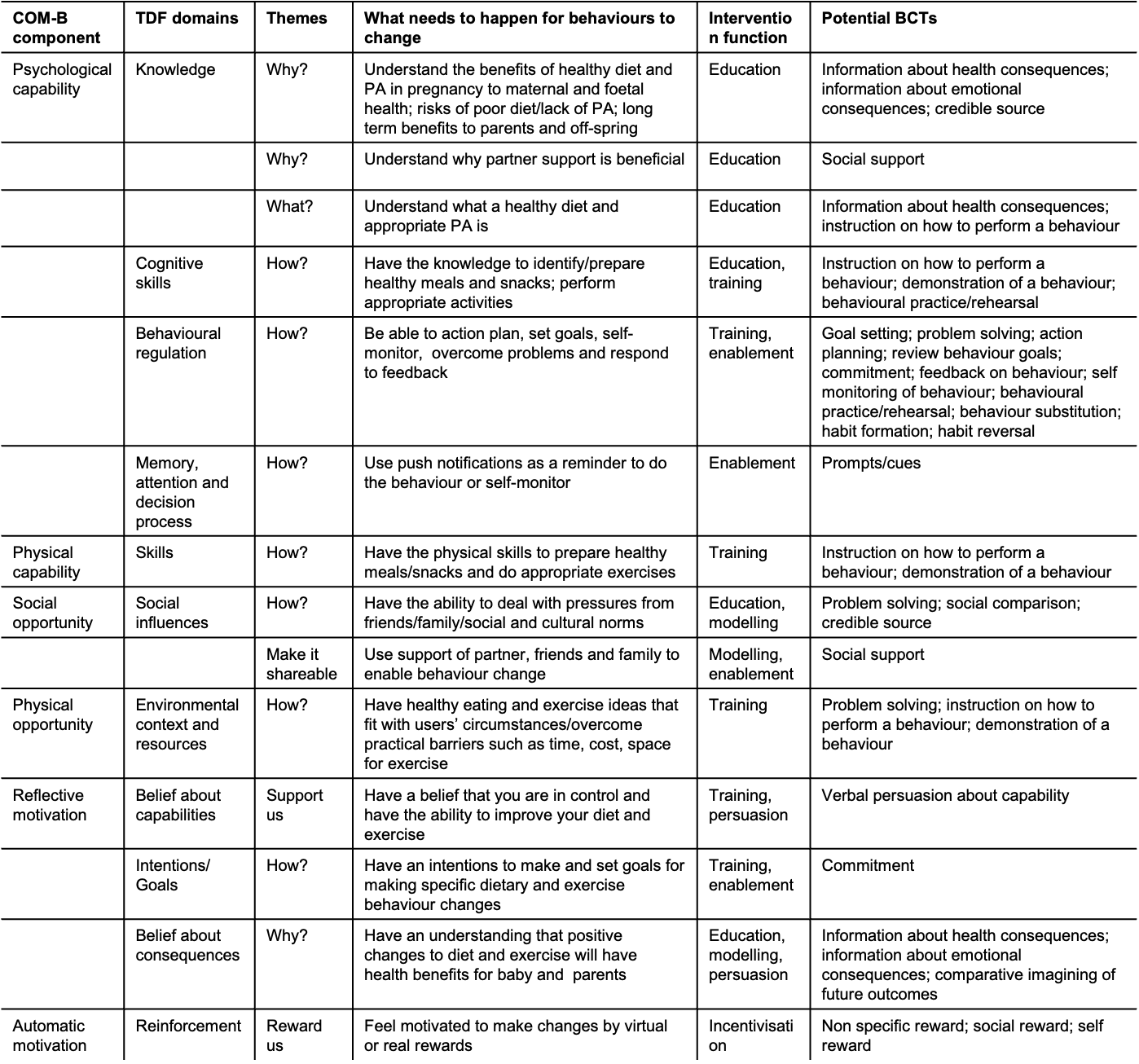

Supplement: Multimedia Appendix 1 [file formative_v7i1e44082_app1.docx]
