# Supplementary material for: Using the Person-Based Approach to Develop a Digital Intervention Targeting Diet and Physical Activity in Pregnancy: Development Study
Source: JMIR Form Res. 2023 May 26;7:e44082. doi: 10.2196/44082 (PMC10257111; doi:10.2196/44082)
Supplement: Multimedia Appendix 2 [file formative_v7i1e44082_app2.docx]

**Multimedia Appendix 2. Examples of bite-size messages**

**Gestation week 6**

**Motivation or challenge:** Make your healthy changes for pregnancy into healthy changes for life.

**Dietary message:** Eating well is important for your pregnancy health and your baby's growth and development. The Eat Well in Pregnancy guide will help you and your baby get the nutrients and goodness you both need. Top Tip: Stick a picture of the guide on your fridge or cupboard as a reminder.

**Physical activity message:** Off you go! Watch this video about activity in pregnancy - it's full of important information on why keeping active is important and how exercise safely (APF intro video to be embedded) Top Tip: Many people find that tracking their steps on their phone helps motivate them to move more. You could download Active 10 - a free NHS approved app to help you stay moving! Search Active10 in the Appstore or on Google Play.

**Extra message:** Are you drinking enough? (no, we aren't talking alcohol here). Drinking plenty of water when you're pregnant is important for your and baby's health and it helps with common pregnancy problems like tiredness and constipation. Top Tip: Drink a glass of water with every meal - make it a habit by adding it as a baby step.

**Gestation week 13**

**Motivation or challenge:** Active challenge: Make a date with someone for a walk or some other activity this week.

**Dietary message:** Beans and pulses like lentils and chickpeas count as vegetables for one of your 5 a day. Plus they are a great source of protein and fibre. Check out what Dr Giles Yeo has to say about fibre here. Top Tip: Adding beans or lentils to stews, soups and pasta sauces adds goodness and makes your meals stretch further. Check out our ideas here.

**Physical activity message:** It might sound strange, but if you're feeling tired, being active can actually make you feel less tired. Going for a walk or doing a workout releases endorphins - hormones which make you feel good. But build up the amount you do gently. Take a look at these guides for different activities you might enjoy in pregnancy.

**Extra message:** A slow cooker is a great way to make tasty, low-effort meals. They cost from around £30 but they use far less electricity than a regular oven so you could be saving in the long run. Top tip: Slow cookers work really well with cheaper cuts of meat and root vegetables like carrots, swedes and parsnips. Check out our slow cooker recipes here.

**Gestation week 30**

**Motivation or challenge:** Bite size exercise challenge: See if you can fit 5 micro movements into parts of your day when you are normally sitting down. A micro movement is a really quick burst of exercise. For example two countertop push ups, two squats, walking on the spot for a minute or anything else that gets you moving.

**Dietary message:** A happy gut is great for our physical and mental health. Watch Dr Giles Yeo's video about how to keep your gut happy. Top Tip: Our gut loves variety. Aim for different coloured fruit and vegetables in your shopping basket!

**Physical activity message:** How about setting yourself a walking goal for the last 10 weeks of your pregnancy. For example 6000 steps a day adds up to around 300 kilometres over 10 weeks which is the same as walking from Newcastle to Brighton! Top Tip: Plan a 'virtual walk' and track your progress on a map.

**Extra message:** Labour can be hard work (well - it's in the name!). Keeping active in these last few weeks can help to your body ton prepare. And practice breathing exercises as they can help you stay calm in labour.
